# Efficacy and safety of focused low-intensity pulsed ultrasound versus pulsed shortwave diathermy on knee osteoarthritis: a randomized comparative trial

**DOI:** 10.1038/s41598-022-17291-z

**Published:** 2022-07-27

**Authors:** Lang Jia, Dongqian Li, Xia Wei, Jinyun Chen, Deyu Zuo, Wenzhi Chen

**Affiliations:** 1 Department of Rehabilitation Medicine, The Second Affiliated Hospital of Chongqing Medical University, Chongqing, China; 2 State Key Laboratory of Ultrasound in Medicine and Engineering, College of Biomedical Engineering, Chongqing Medical University, Chongqing, China; 3 Department of Rehabilitation, Chongqing Traditional Chinese Medicine Hospital, Chongqing, China; 4 Clinical Center for Tumor Therapy, The Second Affiliated Hospital of Chongqing Medical University, Chongqing, China

**Keywords:** Osteoarthritis, Osteoarthritis

## Abstract

The aim of this study was to compare the efficacy and safety of focused low-intensity pulsed ultrasound (FLIPUS) with pulsed shortwave diathermy (PSWD) in subjects with painful knee osteoarthritis (OA). In a prospective randomized trial, 114 knee OA patients were randomly allocated to receive FLIPUS or PSWD therapy. The primary outcome was the change from baseline in the Western Ontario and McMaster Universities Osteoarthritis Index (WOMAC) total scores. Secondary outcomes included the numerical rating scale (NRS) for pain assessment, time up and go (TUG) test, active joint range of motion (ROM) test, and Global Rating of Change (GRC) scale. Data were collected at baseline, 12 days, 12 weeks and 24 weeks. Patients receiving FLIPUS therapy experienced significantly greater improvements in the WOMAC total scores than patients receiving PSWD therapy at 12 days (mean difference, − 10.50; 95% CI − 13.54 to − 7.45; *P* = 0.000). The results of the NRS, TUG test, ROM test and GRC scale showed that participants treated with FLIPUS reported less pain and better physical function and health status than those treated with PSWD at 12 days (*P* = 0.011, *P* = 0.005, *P* = 0.025, *P* = 0.011, respectively). Furthermore, patients in the FLIPUS group showed significant improvements in the WOMAC total scores and NRS scores at 12 weeks (mean difference, − 7.57; 95% CI − 10.87 to − 4.26; *P* = 0.000 and − 1.79; 95% CI − 2.11 to − 1.47, respectively) and 24 weeks (mean difference, − 6.96; 95% CI − 10.22 to − 3.71; *P* = 0.000 and − 1.37; 95% CI − 1.64 to − 0.96; *P* = 0.000, respectively) of follow-up. There were no adverse events during or after the interventions in either group. This study concluded that both FLIPUS and pulsed SWD are safe modalities, and FLIPUS was more effective than PSWD in alleviating pain and in improving dysfunction and health status among subjects with knee OA in the short term.

**Trial registration:** Chinese Clinical Trial Registry, ChiCTR2000032735. Registered 08/05/2020, http://www.chictr.org.cn/showproj.aspx?proj=53413.

## Introduction

Knee osteoarthritis (OA) is characterized by pathology involving the whole joint, including cartilage degradation, bone remodeling, osteophyte formation, and synovial inflammation, leading to joint pain, stiffness, and swelling and difficulty with purposeful movement and having a considerable impact on health status^1^. The overall prevalence of symptomatic knee OA was 8.1% in China. A total of 5.7% of men and 10.3% of women over 45 years of age have symptomatic knee OA, suggesting that the risk in women is higher than that in men^2^. To date, knee OA is likely to become the eighth most important cause of disability in males and the fourth most important global cause in females.

Currently, effective conservative management is typically limited to the treatment of symptoms until the late stages of arthritis require surgical procedures^3,4^. However, invasive, operative and oral nonsteroidal anti-inflammatory drugs are associated with some potential for adverse outcomes. Therefore, noninvasive, minimum side effect and cost-effective approaches could be applied in the early or middle stages of knee OA, as they are crucial for relieving symptoms, reducing complications, lowering disability rates, improving health status and lessening the economic burden on the health care system and on patients with knee OA. A recent randomized controlled trial reported improvement in pain and functional capacity by the implementation of strengthening exercises in nonweight-bearing positions in knee OA patients^5,6^.

In recent years, the mechanical effects of focused low-intensity pulsed ultrasound (FLIPUS) and the thermal effects of pulsed shortwave diathermy (PSWD) have been therapeutic options for patients with mild to moderate knee OA^7^. Both modalities are aimed at the management of knee OA and are performed in part to relieve pain, increase range of motion (ROM), accelerate tissue repair, reduce edema and disability and, if possible, slow its progression^8,9^ and are therefore conditionally recommended for patients with knee OA by clinical guidelines^10^. Assuming a comparable indication and efficacy of both FLIPUS and PSWD, the question of whether one of the two modalities should be preferred remains unanswered.

Previous studies have directly compared traditional nonfocused therapeutic ultrasound with shortwave diathermy^11,12^. However, there was no significant difference between the two modalities in terms of pain reduction and functional improvement^12^. We speculated that the same biological action (thermal effects) and therapeutic targets (muscles and tendons) of traditional ultrasound and shortwave diathermy led to similar effectiveness in the management of knee OA.

To date, only one result has been obtained regarding FLIPUS in the context of knee OA^9^. No clinical trial has directly compared the mechanical effects of FLIPUS and the thermal effects of PSWD in patients with mild to moderate knee OA. The aim of this study was therefore to compare both the safety and clinical effects of a twelve-day intervention with each device in a population with knee OA.

## Materials and methods

### Study design

This trial was designed according to the CONSORT 2010 statement. This was a prospective, randomized, comparative, observer-blinded study with a 24-week follow-up performed between June 2020 and December 2020.

The study was approved by the institutional review board for human investigation and followed the Declaration of Helsinki, 1996. The study was registered at the Chinese Clinical Trial Registry (ChiCTR2000032735, date of registration: 08/05/2020).

### Participants

Subjects were recruited through advertisements placed in the rehabilitation department of the Second Affiliated Hospital of Chongqing Medical University. The inclusion and exclusion criteria are shown in Table 1. All subjects gave written informed consent before participating in the study. The study was approved by the Ethics Committee of the Second Affiliated Hospital, Chongqing Medical University. The Chinese Clinical Trial Registry granted full approval of the study protocol, recruitment materials, and consent form (URL: https://www.chictr.org.cn; unique identifier: ChiCTR2000032735; date of registration: 08/05/2020). The procedures conformed with good clinical practice and with the ethical standards laid down in the 1964 Declaration of Helsinki and its later amendments.

**Table 1:** Inclusion and exclusion criteria.

**Inclusion criteria**
Aged 40–80 years (either sex)
Met the criteria for the ACR clinical classification of knee OA
Had radiographic evidence of knee OA (weight-bearing views) assessed as Kellgren–Lawrence grade I to grade III
Had average knee pain ≥ 3 on an 11-point NRS in the past week
**Exclusion criteria**
Knee pain caused by other diseases (rheumatoid arthritis, gouty arthritis, infectious arthritis)
A history of knee joint replacement on the study knee; current or past (within 6 months) oral or intra-articular corticosteroid use
Physiotherapy, acupuncture treatment, the use of exercises specifically for the knee within the past 6 months
A medical condition that precludes safe exercise (such as uncontrolled hypertension, a heart condition, hematological diseases, coagulopathy, gastrointestinal ulcers, or hemorrhage)
A history of taking NSAIDs or symptomatic slow-acting drugs for OA (diacerein, hyaluronic acid) within the previous 30 days
The inability to complete the study

*ACR* American College of Rheumatology, *OA* osteoarthritis, *NRS* Numerical Rating Scale, *NSAIDs* nonsteroidal anti-inflammatory drugs.

### Procedures

The study consisted of a screening visit (a detailed medical history, a physical examination, and the collection of weight, sex, age, height, and duration of knee OA data), a baseline visit during which FLIPUS or PSWD was performed, and follow-up visits at 12 days, 12 weeks and 24 weeks posttherapy. There was no treatment after 12 days. Potential study participants returned for a baseline visit after a 1-week washout period for nonsteroidal anti-inflammatory drugs (NSAIDs) and analgesics. Before the treatment, the demographic data and baseline assessments of each patient were initially collected in an interview and evaluated by an attending orthopedist, and the evaluation was then confirmed by a well-trained and experienced research team physiatrist in a separate physical therapy room.

### Randomization procedures

Enrolled patients were randomized (1:1) to 2 groups. Opaque randomization envelopes with sequentially numbered allocations were generated by a person who was not clinically involved in the study. When a patient consented to the trial, the patient selected one of the envelopes and was then given the allocated therapy^13^.

### Intervention

**Figure 1:**
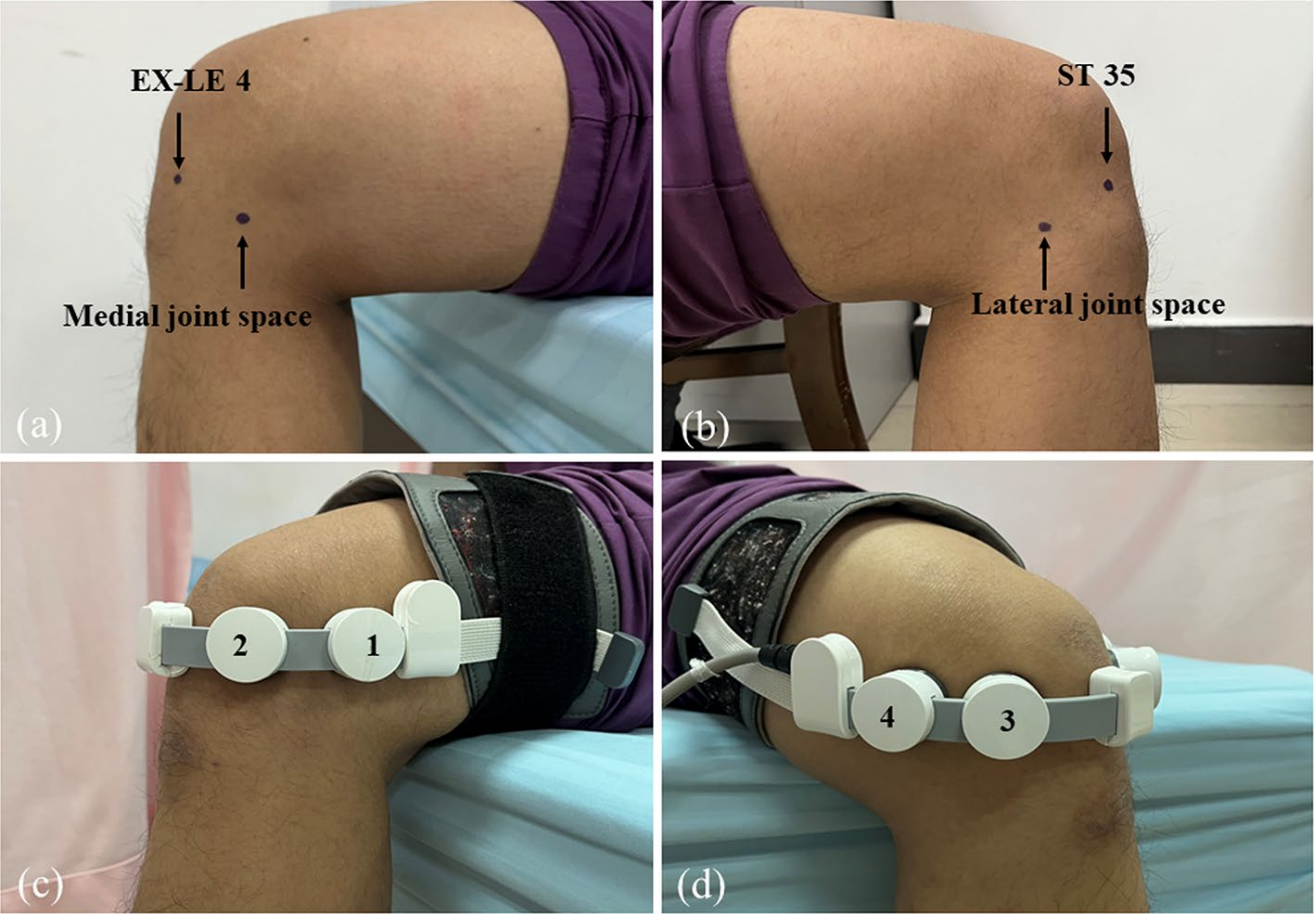
Procedure used for FLIPUS. The ST-35, EX-LE 4, medial and lateral knee joint spaces were marked, and the ultrasonic heads were fixed to ST 35, EX-LE 4, and the knee joint space.

The patients in the FLIPUS group received focused low-intensity pulsed ultrasound therapy (The Model CZG200 Ultrasound Therapeutic Device for Arthritis, Chongqing Haifu Medical Technology Co. Ltd., China) for 20 min once daily for a total treatment duration of 12 days^9^. The device had an ultrasonic transducer diameter of 25 mm, a radius of curvature of 28 mm, a frequency of 0.6 MHz, a pulse repetition frequency of 300 Hz, a spatial and temporal average intensity (Ista) of 120 mW/cm^2^, and a duty cycle of 20%^9^. All treatments were standardized using a device that placed the participant in a sitting position, and the knee was angled ~ 90° in the flexion position. The four ultrasound probes were close to the surface skin of the EX-LE 4 acupoint (located in the depression medial to the patellar ligament when the knee was flexed), ST 35 acupoint (located in the depression lateral to the patellar ligament when the knee was flexed), and medial and lateral knee joint spaces, as previously described^9^ (Fig. 1).

The patients in the PSWD group received pulsed shortwave diathermy therapy (The Curapulse 970 model, Enraf–Nonius, Rotterdam, the Netherlands) for 20 min once daily for a total treatment duration of 12 days^13^. The machine’s program was set at ‘chronic OA model’ and delivered electromagnetic waves with a frequency of 27.12 MHz, a pulse repetition frequency of 300 Hz, a pulse duration of 300 μs, a peak power of 200 W, and a mean power of 18 W^14^. All patients reported a mild but comfortable feeling of warmth.

Additionally, both groups received daily care instructions and core treatment as described in recent randomized controlled trials^5,6,15^, including the provision of accurate verbal and written information to all patients with knee OA to enhance their understanding of the condition and healthcare professionals’ management and information on positive behavioral changes (weight loss, suitable footwear and pacing), local muscle strengthening, and general aerobic fitness^16^.

Patients with symptoms in both knees received treatment for both knees, but trial outcomes were assessed only in the knee with worse symptoms (higher total score using the Western Ontario and McMaster Universities Osteoarthritis Index)^14^.

### Primary outcome measure

The primary outcome measure was functional capacity, assessed by the Western Ontario and McMaster Universities Osteoarthritis Index (WOMAC)^17^. The WOMAC contains 24 items and is composed of three subscales: the pain (5 questions, scores of 0–20), physical function (17 questions, scores of 0–68), and stiffness (2 questions, scores of 0–8) subscales. Each item is rated on a scale of 0 to 10 (with higher scores indicating worse pain, function, and stiffness), and the total score ranges from 0 to 96. The minimal clinically important difference for knee OA is − 7.9 points for the WOMAC total score^18^.

### Secondary outcome measures

The secondary outcomes included the following: (1) Scores on the numerical rating scale (NRS), which assesses knee pain during movement for 5 min (minimal clinically important difference, ≥ 15% improvement from baseline)^19^. The NRS instrument consists of 10-cm horizontal or vertical lines; a score of 0 cm indicates no pain, whereas a score of 10 cm indicates very severe pain. (2) Scores on the Timed Up and Go test (TUG), which assesses knee functional tasks. (minimal clinically important improvement for the TUG is 1.14 s^20^). Subjects were instructed to stand up from a chair, walk 3 m comfortably and safely, come back and sit back down in the chair. The time taken to complete this task was measured with a stopwatch timed to the nearest 1/100 s. (3) Active ROM scores, which were measured with a goniometer with 30-cm movable double arms, marked in 1-degree increments. Knee flexion was measured in the supine position, with the foot on the measured side resting on the therapeutic bed as far as possible. The fully extended knee was considered the zero position, and the degrees of maximum flexion, maximum extension, and extension deficit were recorded. A negative ROM score for extension indicated that the patient was unable to reach the zero position. The angle between maximum flexion and maximum extension was described as the excursion range. (4) Scores on the Global Rating of Change (GRC) scale, which assesses a self-perceived change in health status (minimal clinically important difference, a score of + 3 or higher^21^). The scores range from − 7 to + 7, with higher positive values indicating more improvement and lower negative values indicating worsening symptoms. All patients were evaluated at baseline, after 12 days of treatment, and at follow-up after 12 and 24 weeks.

Safety measures assessed during the treatment phase included treatment-emergent adverse events (TEAEs), serious adverse events (SAEs), assessments of vital signs, severity of pain, swelling, feelings of vasodilatation and subcutaneous bruises due to adverse events.

### Sample size

Case number planning was calculated for the primary outcome measure and the reduction in the WOMAC total score compared to baseline. Based on a study by Tubach et al.^18^, who found a reduction of 7.9 points in the WOMAC total score (effect size of 0.59), the case number was calculated using a t test-based model in G * Power version 3.1.5^22^. A power of 0.8 with a significance level of 0.05 was chosen and resulted in a required number of 47 participants. We added approximately 10–20% more participants to account for potential loss to follow-up, resulting in a final enrollment goal of 114 participants (57 per group).

### Statistical analysis

Independent t tests (continuous variables), Mann–Whitney U tests (ordinal variables), and chi-square tests (nominal variables) were used to compare the demographics, clinical features of patients, and primary and secondary outcomes of the FLIPUS group and PSWD group.

All outcomes were analyzed using the intention-to-treat approach. We tested the data normality and sphericity using the Shapiro–Wilk test and Mauchly’s test, respectively. Repeated measures analysis of covariance (ANCOVA) was used to compare the WOMAC score and Numerical Rating Scale score at baseline, after 12 days of treatment, and at follow-up after 12 and 24 weeks, as well as the Timed Up and Go test, joint ROM test, and GRC scale scores at baseline and after 12 days of treatment.

Post hoc analyses were conducted within (using paired t tests for comparisons across time) and between (using independent t tests within each time point) groups via pairwise comparisons with Bonferroni correction. Repeated measures ANCOVA using a last-observation-carried forward (LOCF) approach for the imputation of missing data was applied as a supportive analysis.

All statistical analyses were performed using SPSS version 20 (SPSS Inc., Chicago, IL, USA) with a global alpha of 0.05.

## Results

### Patient characteristics

**Figure 2:**
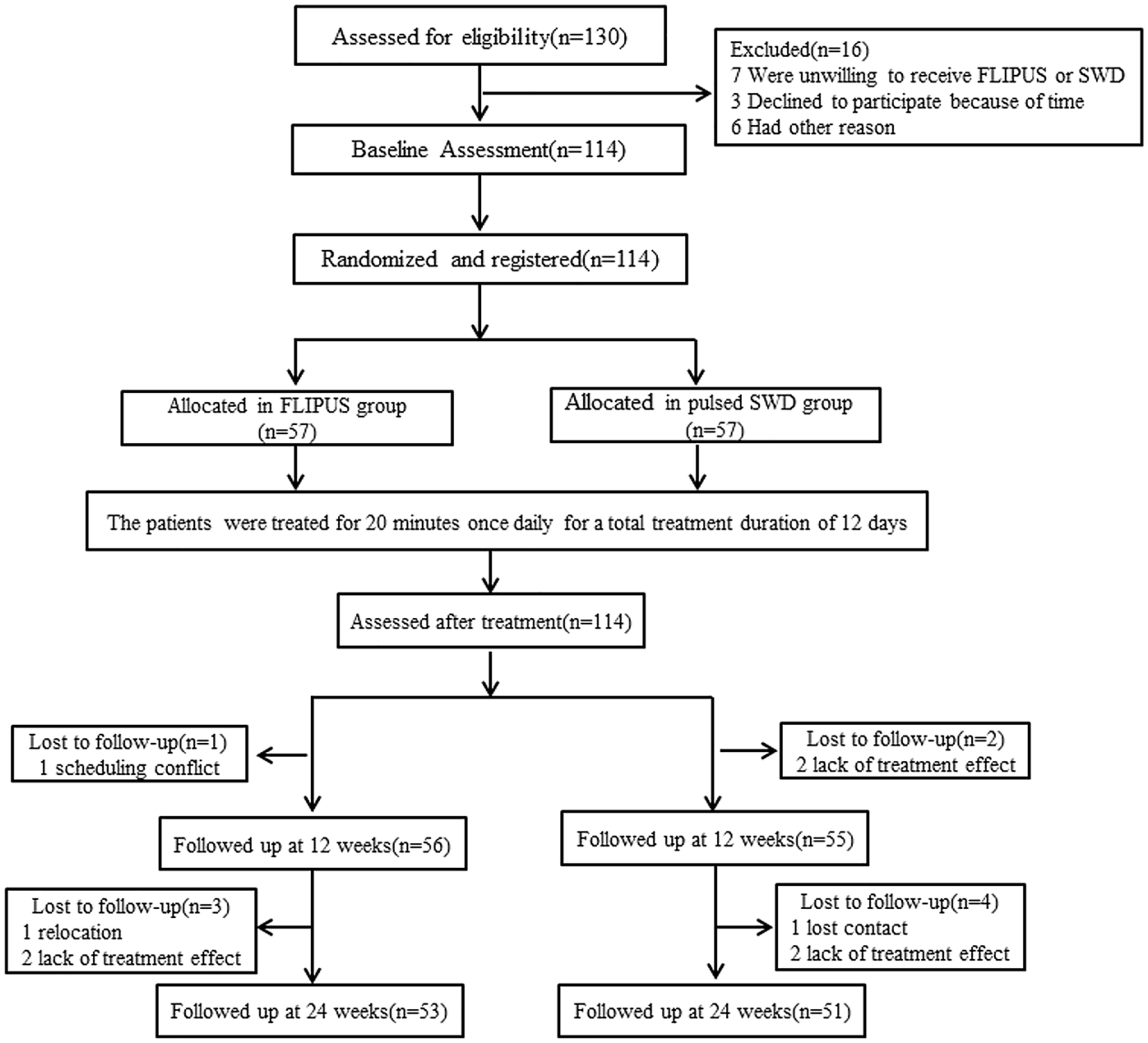
Flowchart of eligible and recruited participants (CONSORT diagram). FLIPUS: focused low-intensity pulsed ultrasound; PSWD: pulsed shortwave diathermy.

A total of 130 patients were screened in this study. After 16 patients were excluded, 114 were evenly distributed between the two arms. A total of ten (8.8%) patients dropped out during the trial: four (7.0%) in the FLIPUS group and six (10.5%) in the PSWD group (Fig. 2).

The baseline characteristics of the sample are shown in Table 2. No differences were found between the groups at the beginning of the trial. There were no losses during the intervention period.

**Table 2:** The baseline demographics and clinical features of patients.

Variable	FLIPUS (n = 57)	PSWD (n = 57)	*P* value
Age, years	62.28 ± 10.88	59.93 ± 8.97	0.211
Women, no. (%)	42 (73.68%)	45 (78.95%)	0.660
BMI, kg/m^2^	25.18 ± 3.26	25.29 ± 2.85	0.854
Duration of disease, months	120.32 ± 74.88	118.54 ± 89.73	0.909
**Kellgren–Lawrence grade, no. (%)**			
Grade I	4 (7.01%)	6 (10.53%)	0.205
Grade II	40 (70.18%)	45 (78.94%)	
Grade III	13 (22.81%)	6 (10.53%)	
**Primary outcome**			
WOMAC			
Total	34.49 ± 10.26	33.70 ± 7.91	0.646
Pain	6.40 ± 2.15	7.44 ± 2.61	0.551
Stiffness	2.35 ± 1.13	2.56 ± 1.02	0.558
Physical function	23.89 ± 8.37	25.54 ± 6.58	0.688
**Secondary outcomes**			
NRS score	5.51 ± 1.28	5.81 ± 1.06	0.825
GRC score	− 2 (2)	− 1 (3)	0.066
ROM, degree	127.39 ± 5.92	126.23 ± 6.57	0.895
TUG test score	12.91 ± 2.45	12.60 ± 2.33	0.296

*FLIPUS* focused low-intensity pulsed ultrasound, *PSWD* pulsed shortwave diathermy, *BMI* body mass index, *WOMAC* Western Ontario and McMaster Universities Osteoarthritis Index, *NRS* Numerical Rating Scales, *GRC* Global Rating of Change scale, *IQR* interquartile range, *ROM* range of motion; *TUG* Timed Up and Go test. Measurement data are represented as the mean ± SD (normally distributed data) or median (IQR) (nonnormally distributed data). Enumeration data are represented as frequencies (proportions).

### Primary outcomes

**Figure 3:**
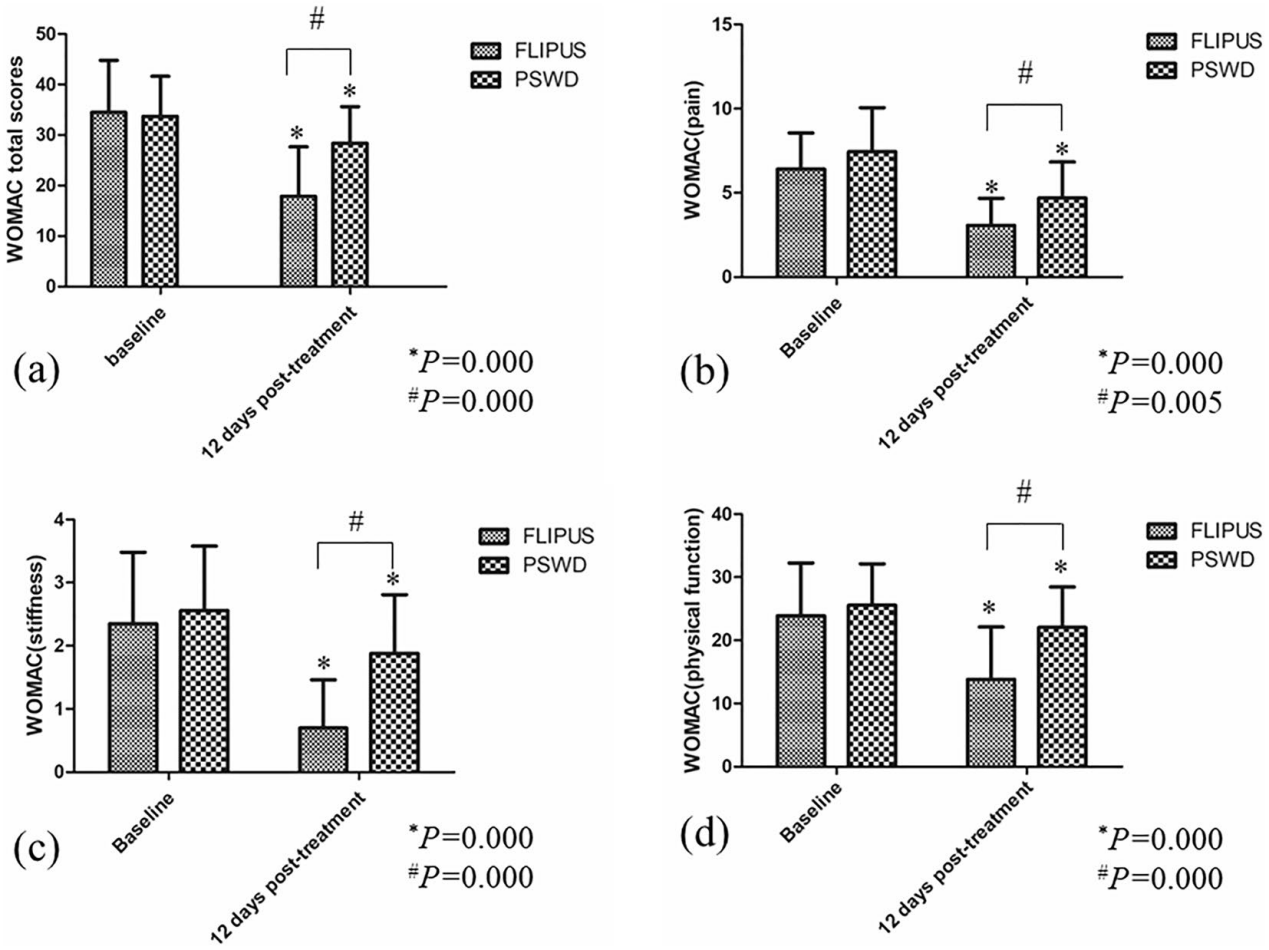
Primary efficacy measures after 12 days of treatment. (**a**) WOMAC total score, (**b**) pain subscore, (**c**) joint stiffness subscore, and (**d**) physical function subscore. Error bars indicate standard deviations. *indicates a significant within-group difference between pretreatment scores. ^#^indicates a significant between-group difference after 12 days of treatment.

FLIPUS-treated patients experienced significantly greater pain reduction and improvements in physical function and stiffness than PSWD-treated patients according to the WOMAC total and subscale scores after 12 days of treatment (*P* = 0.005, *P* = 0.000, *P* = 0.000 and *P* = 0.000, respectively) (Fig. 3a–d). Additionally, there was a statistically significant improvement in both groups according to the WOMAC total scores after 12 days of treatment compared with the baseline scores (Fig. 3a). However, the PSWD group did not exhibit an improvement of − 7.9 points from baseline in the WOMAC total score after 12 days of treatment. Specifically, 4 patients (7.02%) in the FLIPUS group and 22 patients (38.60%) in the PSWD group failed to reach the minimal clinically important difference for the WOMAC total score. In addition, the WOMAC total score remained significantly lower at the 12-week and 24-week follow-ups than at baseline in the FLIPUS group but was higher at the 24-week follow-up than at baseline in the PSWD group (*P* = 0.000; Table 4).

### Secondary outcomes

#### NRS score

Patients in the FLIPUS group showed better analgesia effects than those in the PSWD group after 12 days of treatment (*P* = 0.011; Table 3). A significantly greater percentage of FLIPUS-treated patients, compared to PSWD-treated patients, experienced a greater reduction in pain from baseline according to the NRS score. Both groups showed an improvement of at least 15% from baseline according to the NRS scores after 12 days of treatment. In addition, the NRS score remained significantly lower at the 12-week and 24-week follow-ups than at baseline in the FLIPUS group but was higher at the 24-week follow-up than at baseline in the PSWD group (*P* = 0.000; Table 4).

**Table 3:** Secondary efficacy measures after 12 days of treatment.

Variable	FLIPUS	PSWD	Mean between-group difference: FLIPUS-PSWD (95% CI)	*p* value
n	57	57		
NRS score	1.89 ± 1.01	2.65 ± 1.01	− 0.76 (− 1.12, − 0.39)	0.011
Change (95% CI) from baseline	− 3.61 (− 3.85, − 3.37)	− 3.16 (− 3.36, − 2.95)		
GRC score	+ 4 (2)	+ 3 (2)		0.011
ROM, degree	130.56 ± 4.65	128.30 ± 6.24	2.26 (0.21,4.32)	0.025
Change (95% CI) from baseline	3.16 (2.41, 3.94)	2.07 (1.50, 2.64)		
TUG test score	10.61 ± 2.29	11.84 ± 2.42	− 1.23 (− 2.02, − 0.44)	0.005
Change (95% CI) from baseline	− 2.30 (− 2.63, − 1.97)	− 0.75 (− 0.91, − 0.60)		

*FLIPUS* focused low-intensity pulsed ultrasound, *PSWD* pulsed shortwave diathermy, *NRS* Numerical Rating Scales, *GRC* Global Rating of Change scale, *IQR* interquartile range, *ROM* range of motion; *TUG* Timed Up and Go test, *CI* confidence interval. Measurement data are represented as the mean ± SD (normally distributed data) or median (IQR) (nonnormally distributed data).

**Table 4:** WOMAC total score and NRS score measures after 12 and 24 weeks of follow-up.

	WOMAC (Total scores)				NRS scores			
	Baseline	12 weeks	24 weeks	*P* value	Baseline	12 weeks	24 weeks	*P* value
FLIPUS	34.49 ± 10.26	22.89 ± 9.32	28.02 ± 9.29	0.000	5.70 ± 1.24	3.05 ± 0.99	4.95 ± 0.89	0.000
PSWD	33.70 ± 7.91	30.46 ± 6.77	34.98 ± 6.72	0.000	5.61 ± 1.13	4.84 ± 0.94	6.25 ± 1.01	0.000
*P* value	0.646	0.000	0.000		0.825	0.000	0.000	

*FLIPUS* focused low-intensity pulsed ultrasound; *PSWD* pulsed shortwave diathermy, *WOMAC* Western Ontario and McMaster Universities Osteoarthritis Index, *NRS* Numeric Rating Scales. Measurement data are represented as the mean ± SD (normally distributed data).

#### Knee ROM

There was a significant difference between the two groups with respect to the range of knee motion after 12 days of intervention (*P* = 0.025; Table 3), and the total mean increment of range of knee motion was greater in the FLIPUS group than in the PSWD group (*P* = 0.000; Table 3).

#### TUG test

Patients in the FLIPUS group performed better knee functional tasks (had lower mean times) than patients in the PSWD group according to the TUG test (*P* = 0.005; Table 3). Notably, the FLIPUS group showed an improvement of at least 1.14 s in the TUG test score from baseline after 12 days of treatment. However, the PSWD group failed to reach a clinically important improvement according to the TUG test.

#### GRC scale

Patients in the FLIPUS group had a better health status than patients in the PSWD group with respect to the GRC scale after 12 days of intervention (*P* = 0.011; Table 3). However, both groups’ scores were considered to be clinically meaningful according to the GRC scale after 12 days of treatment. In addition, a total of 15 patients (26.32%) in the FLIPUS group, compared with 26 (45.61%) in the PSWD group, did not have a score of + 3 or higher on the GRC scale after 12 days of treatment (Table 3).

#### Adverse events

No adverse effects (mild pain, swelling and feeling of vasodilatation, subcutaneous bruises, etc.) were observed in either group in the present trial.

## Discussion

The aim of this study was to compare FLIPUS with PSWD in symptomatic patients with clinical and radiographic evidence of knee OA, and the results showed that the immediate posttreatment pain relief expected with PSWD therapy can also be obtained with FLIPUS therapy; however, pain relief and physical function improvements from FLIPUS therapy persisted for 24 weeks. We performed sensitivity analyses and subgroup analyses, and the results did not favor PSWD in the improvement of physical function. To the best of our knowledge, this is the first study to compare the therapeutic effects of FLIPUS with PSWD on patients with knee OA.

To date, only a few and partly controversial results have been obtained for both ultrasound and PSWD therapy in the context of knee OA. In previous studies, unfocused continuous ultrasonic waves with frequencies of 1 or 1.5 MHz^14–19^ and 1–2.5 W/cm^2^^23–28^ were applied for tendons and muscles around the knee joint^24,26,28^. The biological effects of ultrasound are mainly considered to be thermal, which promotes blood circulation and alleviates spasms in various muscles and tendons^27^. In the last decade, studies have demonstrated that PSWD may also induce an elevation of tissue temperature that is dependent on the total average power delivered^29,30^ and significant physiological thermal effects on blood volume and skin temperature, which may induce vasodilatation, elevate the pain threshold, reduce muscle spasm, accelerate cellular activity, and increase soft tissue extensibility^31^.

Unsurprisingly, the same biological action (thermal effects) and therapeutic targets (muscles and tendons) of unfocused continuous ultrasound and PSWD therapy led to similar effectiveness in the management of knee OA, which may explain why no significant differences were found between unfocused continuous ultrasound and shortwave diathermy^11,12^. However, the thermal effects of ultrasound and PSWD for alleviating muscle and tendon spasms around the joints are insufficient. Muscle does not absorb energy well because of its homogeneity, high water content, and low collagen content, and heating muscles and tendons involves treating a larger area than unfocused ultrasound treats; PSWD can heat effectively^27^, which may explain why only a temporal pain reduction effect was found after ultrasound or PSWD application.

Recently, our previous in vivo experiment^32^ demonstrated that the main biological effects of FLIPUS are mechanical, which could be quite different from the unfocused continuous ultrasound effects reported in other previous studies (thermal effects). In addition to the different biological effects of FLIPUS, selecting the targets for energy application is another key consideration in treatment. We also revealed that FLIPUS at 0.6 MHz could propagate through the patella and soft tissue to stimulate the cartilage directly, which could be quite different from the therapeutic targets of unfocused continuous ultrasound and PSWD reported in other previous studies (tendons and muscles around the knee joint). The potential mechanism of FLIPUS includes promoting extracellular matrix preservation, decreasing the joint effusion volume, proinflammatory mediators, and cell apoptosis, and inducing cell proliferation^32^.

In the current study, the NRS pain scores improved in both groups, which is consistent with an earlier trial, a systematic review and a meta-analysis^9,33^. However, the reduction in the NRS scores was greater in the FLIPUS group than in the PSWD group after 12 days of intervention and during the follow-up period. In addition, the PSWD group failed to reach the minimal clinically important difference at the 12-week and 24-week follow-ups, which indicates that the pain reduction effect in the PSWD group was primarily noticed immediately posttreatment, and the analysis yielded no short-term follow-up effect.

The improvement in the WOMAC total score from baseline failed to reach the minimal clinically important difference in the PSWD group. Furthermore, although the improvements in physical function and disability status according to the TUG test and range of knee motion test were different in both groups after treatment compared with baseline, the improvement in the TUG test in the PSWD group failed to reach the minimal clinically important difference. Based on the present results, PSWD does not improve physical function or disability status in patients with knee OA, which is consistent with an earlier trial^20^. We hypothesize that the improved function and disability were attributable to the better analgesia effects of ultrasonic waves than those of PSWD.

With respect to the screening of self-perceived changes in health status, the median scores on the GRC scale in both groups were above the clinically meaningful threshold of a perceived improvement, which indicated that both modalities could improve health status in symptomatic patients with knee OA. However, FLIPUS showed better improvements in health status than PSWD.

No adverse effects occurred during or after FLIPUS and PSWD treatment in the current study; therefore, both modalities can be used safely in patients with knee OA.

Clinical trials support established daily care and interventions, such as lifestyle modifications (weight reduction and footwear), aerobic or strengthening exercises, physical modalities (transcutaneous electrical nerve stimulation devices) and knee bracing to reduce pain and disability in patients with knee OA. The current study proposed the possibility of using FLIPUS as an adjunct to the current standard of daily care and interventions in the treatment of OA knee pain and dysfunction^16,34–36^. Because of the safety, efficacy and convenience of FLIPUS, it has great potential as a home-based physiotherapy for patients with knee OA, particularly given the need for self-treatment options outside of clinical care that the pandemic has brought upon elderly populations.

The present study has some limitations that should be cautiously considered. First, in the current study, a small number of patients from a single center were included, and these patients were relatively young and had a low BMI. The consequences of this selective bias may be false-negative studies. Therefore, future studies with larger populations, multicenter clinical trials and crossover study designs are needed. Second, several issues remain to be addressed, such as the effects of FLIPUS or pulsed shortwave diathermy on structural changes in the knee joint. Third, while the standard of daily care and interventions are currently supported, further clinical trials are required to confirm the long-term efficacy and safety of a combination of FLIPUS and the current therapeutic regimen in patients with clinical and radiographic evidence of knee OA. Finally, we did not test OA biomarkers in synovial fluid. Bone morphogenetic protein-2, CTX-II and cartilage oligomeric matrix protein may be promising biomarkers for predicting the prognosis and progression of OA and should be studied after intervention.

In conclusion, the current study revealed that both FLIPUS and PSWD are safe treatment modalities, and FLIPUS was more effective than PSWD in improving pain relief, dysfunction and health status among subjects with knee OA.

## Data Availability

All data generated or analyzed during this study are included in this article. Further enquiries can be directed to the corresponding author.
